# Pandemic to endemic: a longitudinal multinational study on COVID-19’s effects on graduate medical education

**DOI:** 10.1186/s12909-026-09657-y

**Published:** 2026-06-12

**Authors:** Fatima Msheik-El Khoury, Jolene Ee Ling Oon, Lorraine Lewis, Halah Ibrahim, Sophia Archuleta

**Affiliations:** 1 Department of Anesthesiology and Pain Medicine, American University of Beirut, Beirut, Lebanon; 2 Division of Infectious Diseases, Department of Medicine, National University Hospital, National University Health System, 1E Kent Ridge Road, NUHS Tower Block, Level 10, Singapore, 119228 Singapore; 3 Department of Medicine, Yong Loo Lin School of Medicine, National University of Singapore, Singapore, Singapore; 4 Accreditation Council for Graduate Medical Education-International, Chicago, IL USA

**Keywords:** Graduate medical education, Residency, COVID-19 pandemic, Resilience, International medical education

## Abstract

**Background:**

This study applied a resilience engineering framework to understand how international graduate medical educations (GME) programs responded to the disruptions caused by the COVID-19 pandemic. Beyond describing early pandemic disruptions, we offer a multi-year, multi-country analysis of how training functions adapted, which elements durably changed, and what program design features may be associated with recovery.

**Methods:**

We conducted a retrospective longitudinal study of all 180 ACGME International (ACGME-I)–accredited programs across seven countries. Annual surveys were completed over three years (Year (Y)1 = Academic Year (AY)2021–22, Y2 = AY2022–23, Y3 = AY2023–24), assessing clinical experiences, educational activities, telemedicine use, and training modifications. Data were analyzed using mixed-effects and generalized linear mixed models, with exact logistic regression for small strata. Subgroup analyses compared specialty, training type, and World Bank income classification. Studying ACGME-I programs provides a common competency-based context, allowing us to describe trajectories of disruption, recovery, and persistence.

**Results:**

Of 180 programs, most were residencies (68.3%) and medical specialties (53.3%), with 87.2% located in high-income countries. Clinical experiences were most disrupted in Y1, especially ambulatory continuity clinics (estimated marginal means [EMM] 1.489) and ambulatory rotations (1.571), both of which recovered by Y3 (1.863 and 1.874; *p*<.001). Inpatient admissions showed similar recovery (1.915→2.120; *p*<.001), while urgent procedures declined slightly (2.013→1.897; *p*=.007). Emergency and ICU exposures initially increased (2.242 and 2.342) but returned toward baseline by Y3 (2.232; 2.244; both *p* > .05). Educational activities were most disrupted for in-person didactics, which nearly ceased in Y1 (0.784) but improved by Y3 (1.386; *p*<.001), offset by a surge in live virtual conferences (2.821→2.476; *p*<.001). Elective rotations also declined (1.440→1.782; *p*<.001), whereas scholarly activity (1.836→1.956; *p*=.002) and direct observation (1.879→1.954; *p*=.005) showed slight disruptions and modest increases by Y3, while one-to-one advising (1.970→2.012; *p* > .05) remained stable. Telemedicine use peaked in Y1 (clinical care 1.020; supervision 0.544) and declined by Y3 (0.720; 0.386; both *p*<.001) but remained in use across programs. Training extensions declined substantially from 43.2% in Y1 to 23.0% in Y3 (OR = 0.11; *p*=.002). Differences by specialty and country income suggest heterogeneous recovery pathways rather than a uniform “return to normal.”

**Conclusions:**

International GME programs demonstrated resilience, restoring clinical and educational activities, integrating telemedicine, and markedly reducing training extensions. Our longitudinal, multi-country data move the field beyond single-site, early pandemic reports by identifying which disruptions resolved, which practices persisted, and which gaps remained. Because all programs shared competency-based structures, findings suggest that specific design features may support adaptation. These results refine the prevailing “back to business as usual” narrative in international graduate medical education by showing a selective reversion with durable innovations.

**Supplementary Information:**

The online version contains supplementary material available at 10.1186/s12909-026-09657-y.

## Background

The COVID-19 pandemic disrupted health systems globally, including graduate medical education (GME) programs. The rapid influx of critically ill patients placed unprecedented pressure on clinical services and led to substantial changes in residency training. Residents experienced redeployments, reduced specialty caseloads, and a rapid shift to virtual platforms for both patient care and educational activities [1]. The disruptions were most pronounced during the initial year of the pandemic and prompted major shifts in the structure and delivery of GME from 2020 onwards.

Early studies on the impact of COVID-19 on residency training have highlighted the adaptability and resourcefulness of GME systems [2, 3]. Residents demonstrated resilience through collaborative problem-solving and innovation, often acquiring new skills in communication, telemedicine, and interprofessional teamwork [2, 3]. While decreased clinical exposure and traditional didactic time raised concerns, these losses may have been balanced by the introduction of technology-enhanced learning and other educational innovations.

Most of the existing literature has focused on the early stages of the pandemic [4], with limited data on its long-term effects on training. Research findings have been mixed, with some studies reporting negative educational outcomes and others emphasizing the benefits of innovation and flexibility. Studies exploring specific adaptations, such as virtual simulations, remote assessments, and telehealth integration, are often limited by their short-term follow-up and a lack of data on feasibility and sustainability [5]. In addition, most studies come from North American and European contexts, with few accounts from international GME settings such as those in the Middle East and Asia, where training occurs within distinct cultural, regulatory, and economic environments [4]. Accordingly, the field lacks comparative evidence on what actually persisted versus reverted as pandemic pressures eased, and how shared program structures shaped those trajectories across countries.

To address these gaps, our study applied a resilience engineering lens to examine how international GME programs adapted over time. Resilience, in this context, is defined not merely as the ability to recover, but as the capacity of a system to adapt, learn, and continue functioning under stress- not by resisting disruption, but by evolving through it. This framework shifts attention from disruption alone to how core training functions are maintained, substituted, or restored over time. It is helpful in the context of pandemic-era GME because it permits examination of both recovery and durable change, rather than treating all departures from pre-pandemic practice as educational loss. Applying this framework, the study explored patterns of disruption, adaptation, and recovery across a diverse set of training environments. We extend prior work in three ways: (1) by analyzing three consecutive academic years (2021–2024) rather than single snapshots; (2) by including 180 programs across seven countries outside the United States and most of Europe; and (3) by examining programs operating under a common competency-based accreditation framework (ACGME-I), which provides a common context for exploring whether structured oversight may contribute to resilience. Specifically, the study aimed to understand how international GME programs responded to the disruptions caused by the COVID-19 pandemic between 2021 and 2024. It assessed changes in clinical experiences, educational activities, telemedicine use, and training modifications, while also examining whether these patterns differed by specialty and by the World Bank income classification of the country of training. By distinguishing reversible disruptions from durable changes, we interrogate the assumption that GME simply “returned to normal.”

## Methods

### Study design and participants

This study employed a retrospective longitudinal design with repeated measures collected at the training program level over a three-year period (2021–2024). Eligible participants included all programs that were accredited by the Accreditation Council for Graduate Medical Education International (ACGME-I) as of 2021. The ACGME-I was established in 2009 to accredit GME programs implementing competency-based medical education (CBME) outside of the United States. Eligible programs were residency and fellowships located across seven countries in Asia and the Middle East.

### Survey development and data collection

Program Directors in all participating programs complete an annual data update as a routine part of the accreditation process. In 2021, a supplemental survey was developed and included in the annual update to assess the impact of the COVID-19 pandemic on GME training and education activities. A team with expertise in GME and survey design collaboratively drafted the survey, refining items through iterative rounds of discussion. An expert in survey methodology then reviewed the survey to ensure clarity, relevance, and methodologic rigor. Survey items (Supplementary file 1) were organized into five domains.

### Program characteristics

Program characteristics included specialty (categorized as medical, surgical, or hospital-based), training type (residency or fellowship). The survey also collected each program’s country name, which was later grouped by income status (classified as lower-middle income or high income based on World Bank designations). These variables were used as between-program covariates in comparative analyses.

### Clinical Experiences (CE)

The clinical experience domain consisted of six items in which programs rated the impact of the pandemic on different types of patient care exposure: ambulatory continuity clinic visits (CE1), ambulatory-based clinical rotations (CE2), inpatient admissions (CE3), urgent or emergent surgeries and procedures (CE4), emergency department consultations (CE5), and intensive care unit (ICU) coverage (CE6). In the original survey (Supplementary file 1), each item was assessed using a six-point ordinal scale, namely 0 for Cancelled, 1 for Significantly decreased, 2 for Moderately decreased, 3 for No effect, 4 for Moderately increased, 5 for Significantly increased, with an additional Not applicable (NA) option. For analysis and to simplify interpretation, responses were recoded into a four-point ordinal scale, with values of 0 for Cancelled, 1 for Decreased, 2 for No Effect, and 3 for Increased.

### Educational Program activities (EP)

Using the same six-point scale, six items evaluated changes in educational program activities, including in-person didactic conferences (EP1), live virtual conferences (EP2), elective rotations (EP3), scholarly activity (EP4), direct observation of residents or fellows by faculty (EP5), and one-on-one advising sessions (EP6). Responses were again recoded into the same four-point scale mentioned above.

### Telemedicine Use (TU)

Telemedicine use was assessed through two separate variables that captured the maximum proportion of use of telemedicine by residents or fellows to provide clinical care (TU1), and how often faculty used telemedicine to supervise trainees (TU2). Response options reflected percentage ranges on a scale of 0 (none), 1 (1–24%), 2 (25–49%), 3 (50–74%), and 4 (≥ 75%).

### Training Modifications (TM)

Finally, three variables captured training extensions implemented in response to the pandemic. These included whether programs required any residents or fellows to extend their training (TM1; yes/no), which was treated as a binary variable, and the number of trainees affected (TM2; continuous count). Programs were also asked whether they had implemented a “platooning” system (TM3; yes/no), defined as dividing residents into teams that alternate schedules to reduce exposure risk, and also treated as a binary variable.

These domains operationalize the resilience framework at the program level by examining whether core educational functions were disrupted, adapted through alternative modalities, or restored over time. Clinical experiences and educational activities captured continuity of training, telemedicine captured adaptive substitution and innovation in care delivery and supervision, and training modifications captured organizational responses used to preserve trainee progression under stress.

### Data analysis

Descriptive statistics summarized the baseline program’s characteristics. Categorical variables, including specialty group, program type, and income status, were summarized as frequencies and percentages. Baseline characteristics were additionally stratified by country income status to describe subgroup distributions across specialty groups and training types.

To examine changes over time, we reported the frequency and valid percentage for each ordinal and binary variable, and median with interquartile range (IQR) for the continuous count variable, across the clinical experience, educational program, telemedicine, and training modification domains across all three survey years. Valid percentages accounted for missing or “not applicable” responses. In addition, we reported raw descriptive statistics (mean and standard deviation) for continuous outcome measures in each survey year. Survey years were defined as follows: Year (Y)1 = academic year (AY) 2021–2022 [baseline], Y2 = AY 2022–2023, Y3 = AY 2023–2024.

For the primary analysis, ordinal variables were treated as continuous. Linear mixed-effects models were used to estimate mean scores over time with program ID specified as a random effect, and time (survey year) as a fixed effect. Estimated marginal means (EMMs) were reported, and pairwise comparisons were conducted to identify statistically significant changes between time points. Y1 (2021–22) was considered the baseline year against which subsequent years (Y2, Y3) were compared.

Mean scores for clinical experiences and educational programs ranged from 0 (canceled) to 3 (increased). For activities initially reduced, higher scores signaled recovery; for those initially increased, lower scores indicated a return to baseline. For telemedicine use variables, mean scores ranged from 0 (none) to 4 (≥ 75%). Higher mean scores reflected greater use of telemedicine, while a decline in scores over time indicated reduced reliance on telemedicine for clinical care or supervision.

Binary outcomes were analyzed using generalized linear mixed models (GLMMs) with a logit link, again with program ID as a random effect. Pairwise comparisons of estimated odds ratios assessed the likelihood of reporting binary outcomes (e.g., training extension) over time.

For a secondary between-program analysis, we assessed whether baseline program characteristics — such as specialty group, income status, or training type — influenced outcomes. This involved including each characteristic as an additional fixed effect in the mixed models to assess baseline differences at Year 1. We then tested interactions between Time and each program characteristic (e.g., Time × Specialty Group) to determine statistically significant differences in change over time across program types.

All descriptive statistics, mixed-effects models, and GLMM analyses were conducted in SPSS (Version 28). For subgroup analyses of the binary variable training extensions (TM1), several strata had small cell counts and quasi-complete separation. To address this, we used exact logistic regression (Stata 18 Software, exlogit) to obtain stable odds ratios and confidence intervals.

### Ethical approval

Ethics approval was obtained from the National University of Singapore Institutional Review Board (NUS IRB Reference No. NUS-IRB-2023-642) and the American University of Beirut, Lebanon (AUB IRB number SBS-2024-0515), with a waiver for informed consent as data was de-identified and retrospective.

## Results

Of 180 eligible programs, all were included in the analysis. 123 (68.3%) were residency programs, and 57 (31.7%) were fellowships. By specialty, 96 (53.3%) were medical, 48 (26.7%) surgical, and 36 (20.0%) hospital-based programs. Using the World Bank income status classification, most programs 157 (87.2%) were located in high-income countries.

Subgroup analysis by income-status showed that among high-income countries, most programs were medical 87 (55.4%) and residency-based 100 (63.7%). In contrast, programs from lower-middle-income countries were more evenly distributed; 9 (39.1%) were medical, 10 (43.5%) surgical, and 4 (17.4%) hospital-based, and were exclusively residency programs 23 (100%) (Table 1).

**Table 1:** Baseline characteristics of programs (*N* = 180)

	*n* (%*N*)	High-income^d^ *n* (%^┼^)	Low-middle-income^e^ *n* (%^┼^)
Specialty Group			
Medical^a^	96 (53.3%)	87 (55.4%)	9 (39.1%)
Surgical^b^	48 (26.7%)	38 (24.2%)	10 (43.5%)
Hospital-based^c^	36 (20.0%)	32 (20.4%)	4 (17.4%)
Training Type			
Residency	123 (68.3%)	100 (63.7%)	23 (100%)
Fellowship	57 (31.7%)	57 (36.3%)	0 (0%)
Country			
Lebanon	18 (10.0%)	-	-
Oman	16 (8.9%)	-	-
Pakistan	5 (2.8%)	-	-
Qatar	36 (20.0%)	-	-
Saudi Arabia	1 (0.6%)	-	-
Singapore	60 (33.3%)	-	-
United Arab Emirates	44 (24.4%)	-	-
Income Status			
High-income^d^	157 (87.2%)	-	-
Lower-middle-income^e^	23 (12.8%)	-	-

^a^Medical programs include: Allergy and Immunology, Cardiovascular Disease, Child Neurology, Child Psychiatry, Dermatology, Endocrinology, Family Medicine, Gastroenterology, Geriatric Medicine, Hematology, Infectious Disease, Internal Medicine, Neonatal/Perinatal Medicine, Neurology, Nephrology, Medical Oncology, Pulmonary Disease, Rheumatology, Pediatrics, Pediatric Cardiology, Pediatric Gastroenterology, Pediatric Nephrology, Pediatric Pulmonology, and Psychiatry

^b^Surgical programs include: General Surgery, Neurological Surgery, Obstetrics/Gynecology, Ophthalmology, Orthopaedic Surgery, Otolaryngology, Pediatric Surgery, Plastic Surgery and Urology

^c^Hospital-based programs include: Anesthesiology, Emergency Medicine, Oncology, Pathology, Pediatric Radiology, Radiology, Radiation and Transitional Year

^d^High-income status referred to programs from Oman, Qatar, Saudi Arabia, Singapore, an United Arab Emirates

^e^Lower-middle-income status referred to programs from Lebanon and Pakistan

^┼^Percentages in the High-income and Lower-middle income columns are calculated within each income group

Descriptive statistics for each outcome across survey years, including counts and percentages for categorical responses and raw mean (± standard deviation) for continuous measures, are presented in Supplementary Tables 1a-4a alongside model-based estimates to provide additional context for the findings.

### Clinical Experiences (CE)

Clinical exposure was most reduced in ambulatory settings during the first year of the pandemic. The EMM for continuity clinic visits (CE1) was 1.489 at baseline, improving significantly to 1.863 by Year 3 (β = 0.374, *p* < .001). Similar recovery was observed for ambulatory rotations (CE2; EMM increased from 1.571 to 1.874, β = 0.304, *p* < .001) and inpatient admissions (CE3; 1.915 to 2.120, β = 0.205, *p* < .001).

**Fig. 1:**
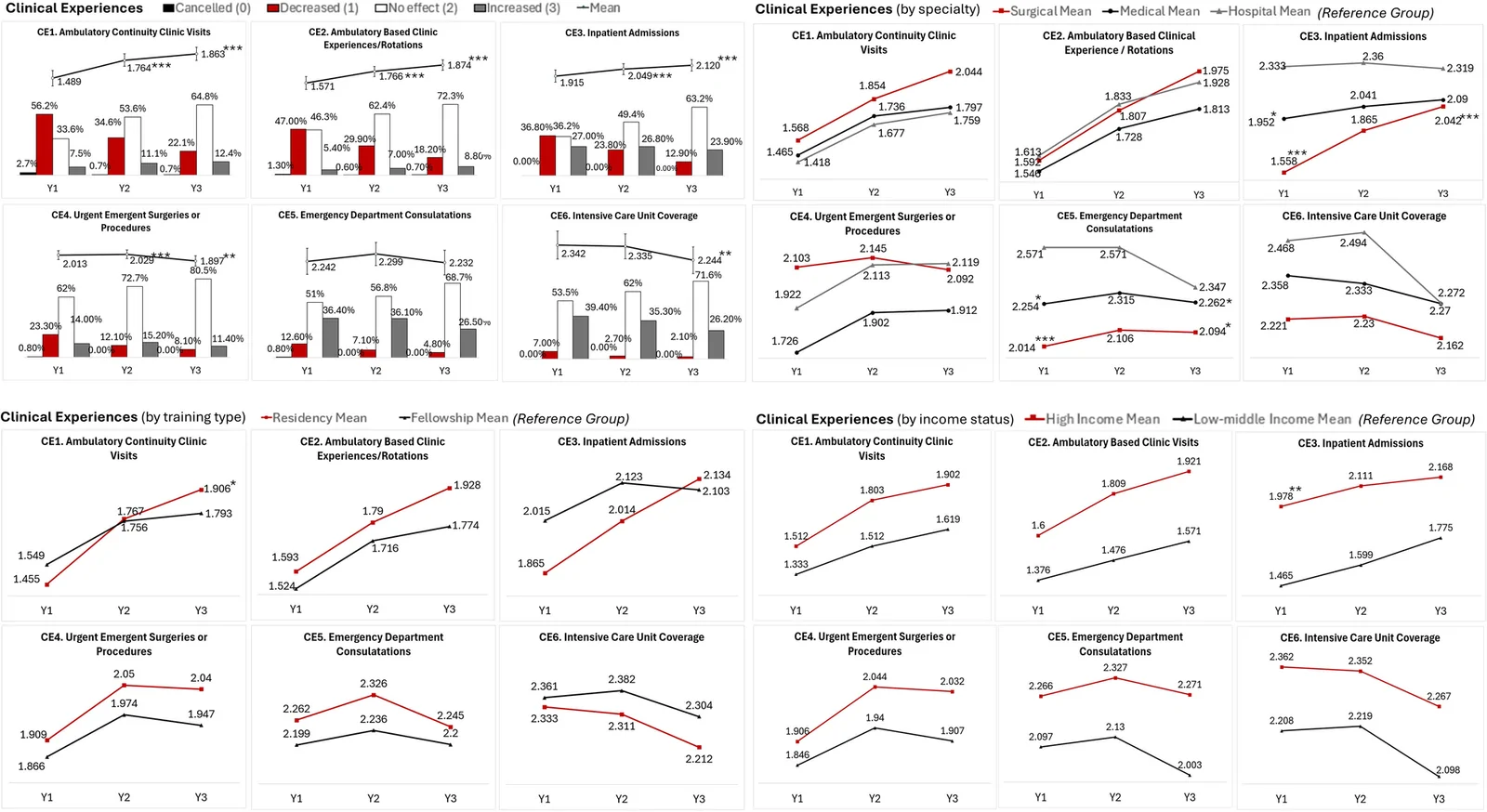
Trends in Clinical Experiences Over Three Years, Stratified by Specialty, Income Status and Training Type Line graphs depict estimated marginal means (EMMs) for six domains of clinical experience (ambulatory continuity visits, ambulatory-based rotations, inpatient admissions, urgent/emergent surgeries, emergency department consultations, and intensive care unit coverage) across three years. Outcomes are stratified by specialty group (surgical, medical, hospital-based [reference]), country income status (high vs. low-middle [reference]), and training type (residency vs. fellowship [reference]). Statistically significant differences in change over time (Year 2 vs. Year 1, Year 3 vs. Year 1), relative to the reference group, are denoted as: *p < .05, **p < .01, ***p < .001

In contrast, exposure to emergent procedures declined significantly (CE4; EMM from 2.013 to 1.897; β = -0.116, *p* = .007), while emergency care (CE5) initially increased and remained elevated by Year 3 (EMM: 2.242 to 2.232; β = -0.010, *p* = .809), and intensive care (CE6) increased initially but declined significantly toward baseline (EMM: 2.342 to 2.244; β = -0.098, *p* = .009) (Fig. 1).

Subgroup analysis showed that hospital-based programs reported significantly higher baseline inpatient admissions (CE3) exposure (EMM = 2.333) than surgical (EMM = 1.558; β = -0.775, *p* < .001) and medical specialties (EMM = 1.952; β = -0.383, *p* = .015), using hospital-based as the reference. Similarly, emergency care (CE5) exposure was higher in hospital-based programs (EMM = 2.571) compared to surgical (EMM = 2.014; β = -0.558, *p* < .001) and medical (EMM = 2.254; β = -0.317, *p* = .021) specialties.

By Year 3, all specialties showed convergence toward baseline. Surgical programs demonstrated the greatest recovery in inpatient admissions (β = 0.497, *p* < .001) and greater stability than hospital-based programs (β = 0.306, *p* = .02). Residency programs showed a significantly steeper recovery in ambulatory continuity clinic visits (CE1) than fellowships (β = 0.207, *p* = .035), though absolute differences were small.

Country income status was associated with baseline inpatient admissions (CE3) exposure: high income programs had higher EMMs (1.978) than lower-middle income programs (1.465; β = 0.513, *p* = .004). This difference diminished over time. Full results for longitudinal trends and subgroup analyses are presented in Supplementary Tables 1a, 1b, 1c.

### Educational Programs (EP)

Educational activities were significantly disrupted in Year 1, with in-person didactics (EP1) showing the greatest reduction (EMM = 0.784). By Year 3, this improved significantly (EMM = 1.386; β = 0.601, *p* < .001). Live virtual conferences (EP2) increased markedly in Year 1 (EMM = 2.821), then declined by Year 3 (EMM = 2.476; β = -0.345, *p* < .001), though usage remained elevated relative to pre-pandemic levels. Elective rotations (EP3) were also substantially affected early (EMM = 1.440) but improved significantly by Year 3 (EMM = 1.782; β = 0.342, *p* < .001).

**Fig. 2:**
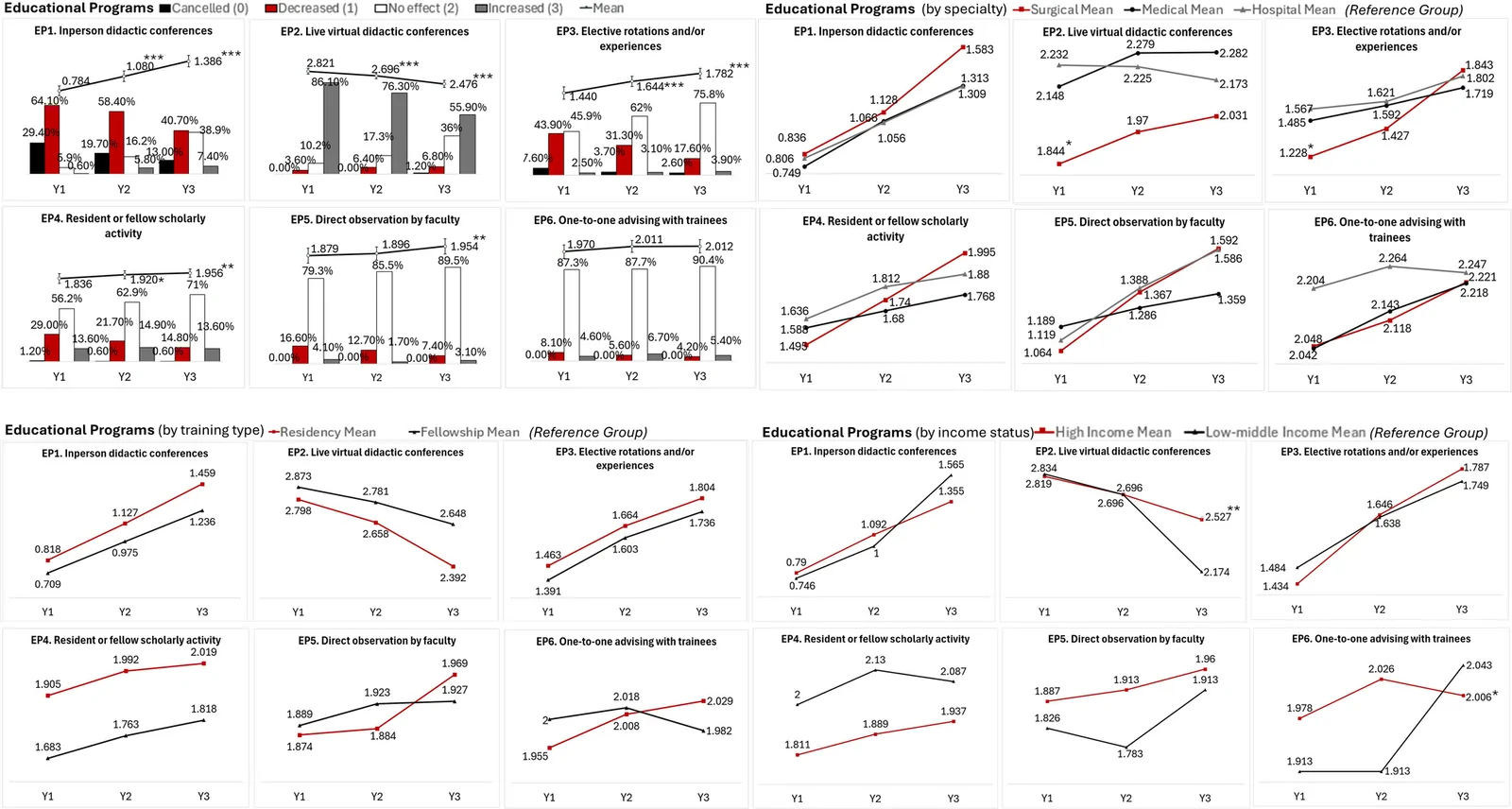
Trends in Educational Activities Over Three Years, Stratified by Specialty, Income Status, and Training Type Line graphs depict estimated marginal means EMMs across three years for educational program activities. Outcomes are stratified by specialty group (surgical, medical, hospital-based [reference]), country income status (high vs. low–middle [reference]), and training type (residency vs. fellowship [reference]). Statistically significant differences are denoted as: *p < .05, **p < .01, ***p < .001

Scholarly activity (EP4) was less disrupted overall (baseline EMM = 1.836), with a modest but significant increase by Year 3 (EMM = 1.956; β = 0.120, *p* = .002). Direct faculty observation (EP5; EMM = 1.879) and one-to-one advising (EP6; EMM = 1.970) were the least affected. EP5 showed a small but significant increase by Year 3 (EMM = 1.954; β = 0.075, *p* = .005), while one-to-one advising remained stable (Year 3 EMM = 2.012; β = 0.024, *p* > .05) (Fig. 2).

Subgroup analysis revealed that, at baseline, surgical programs reported lower live virtual conference (EP2) use than hospital-based programs (EMM: surgical 1.844 vs. hospital-based 2.232; β = -0.388, *p* = .016). Similarly, elective rotation (EP3) exposure was lower in surgical programs (EMM: surgical 1.228 vs. hospital-based 1.567; β = -0.339, *p* = .039). Recovery patterns were similar across specialties, with no significant specialty × time interactions. No significant differences were found between residency and fellowship programs. Country income status did not influence most outcomes, although low-middle income countries showed a significantly steeper decline in EP2 by Year 3 (β = -0.368, *p* = .004). Full detailed results for longitudinal trends and subgroup analyses are presented in Supplementary Tables 2a, 2b, 2c.

### Telemedicine Use (TU)

**Fig. 3:**
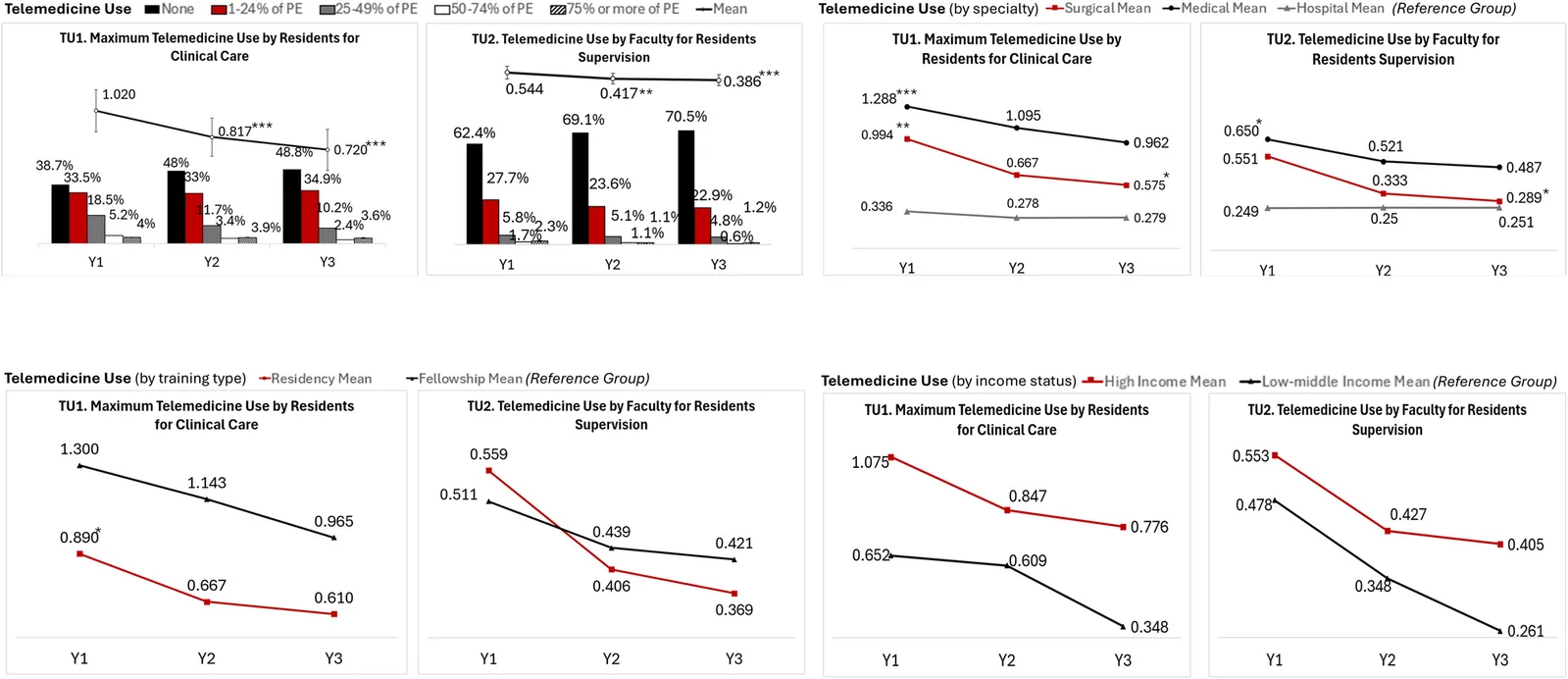
Trends in Telemedicine Use Over Three Years, Stratified by Specialty, Income Status, and Training Type Line graphs depict estimated marginal means (EMMs) across three years for telemedicine use. Outcomes are stratified by specialty group (surgical, medical, hospital-based [reference]), country income status (high vs. low–middle [reference]), and training type (residency vs. fellowship [reference]). Statistically significant differences are denoted as: *p < .05, **p < .01, ***p < .001

Higher TU scores indicate greater telemedicine use. Telemedicine use peaked in Year 1, with resident involvement in clinical care (TU1; EMM = 1.020) and faculty use for supervision (TU2; EMM = 0.544). Both declined significantly by Year 3 (TU1: EMM = 0.720; β = -0.300, *p* < .001; TU2: EMM = 0.386; β = -0.158, *p* < .001) (Fig. 3).

At baseline, medical and surgical programs reported significantly higher TU1 use than hospital-based programs (EMM: surgical 0.994 vs. hospital-based 0.336; β = -0.658, *p* = .005; medical 1.288 vs. hospital-based 0.336; β = 0.953, *p* < .001). For TU2, medical specialties also reported higher use than hospital-based programs (EMM: medical 0.650 vs. hospital-based 0.249; β = 0.401, *p* = .023).

Declines in both TU1 and TU2 were observed across all specialties, with surgical programs showing the most significant reductions by Year 3 (TU1: EMM = 0.575, β = -0.362, *p* = .023; TU2: EMM = 0.289, β = -0.264, *p* = .038). Residents used telemedicine significantly less than fellows at baseline (TU1: EMM = 0.890 vs. 1.300; β = -0.410, *p* = .019), though usage converged by Year 3.

High-income countries reported higher TU1 and TU2 use throughout the study period, but income-based differences were not statistically significant. Full detailed results for longitudinal trends and subgroup analyses are presented in Supplementary Tables 3a, 3b, 3c.

### Training Modifications (TM)

**Fig. 4:**
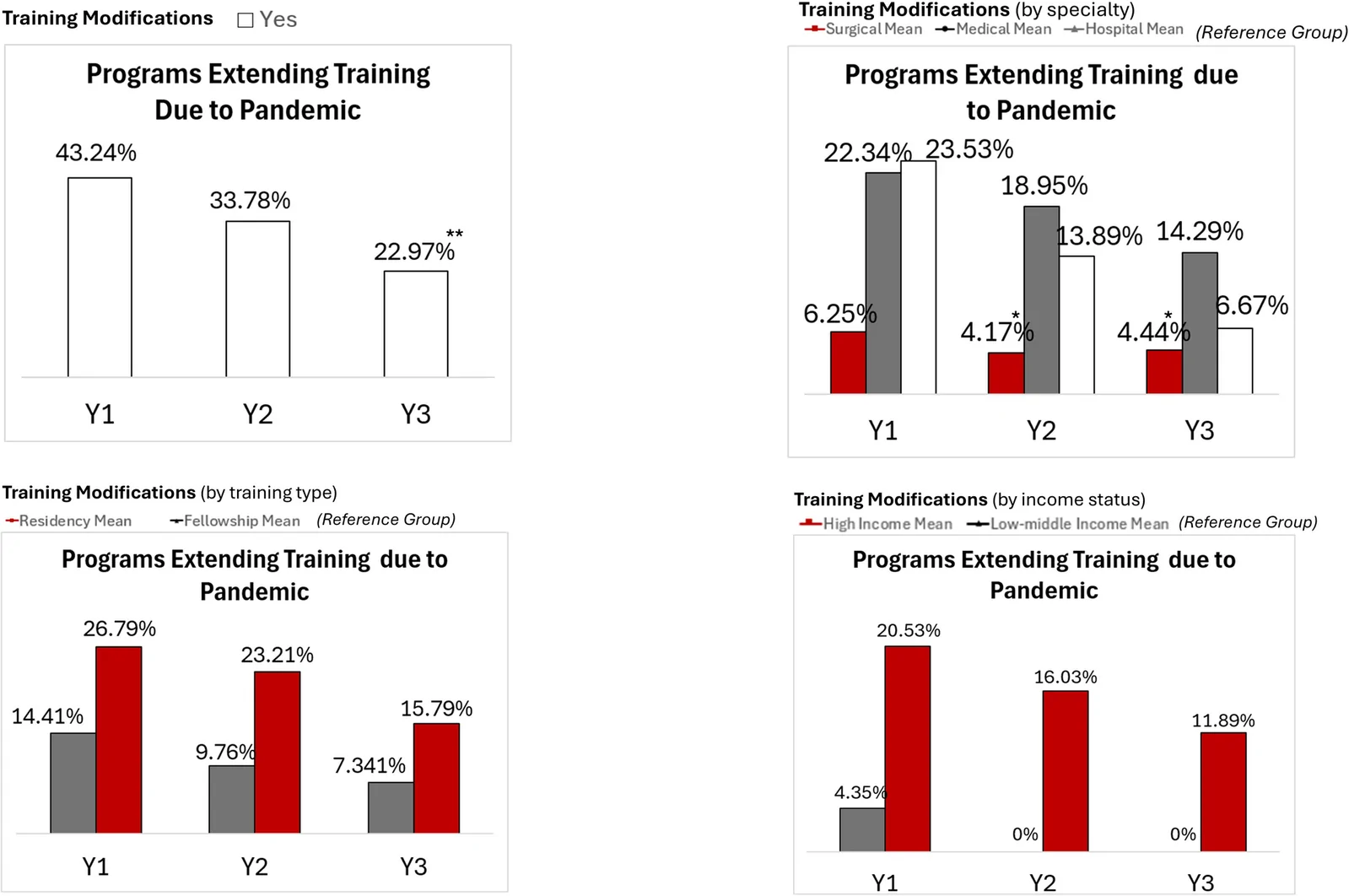
Trends in Training Modifications Over Three Years, Stratified by Specialty, Income Status, and Training Type Bar graphs depict percentages of training modifications across three years. Outcomes are stratified by specialty group (surgical, medical, hospital-based [reference]), country income status (high vs. low–middle [reference]), and training type (residency vs. fellowship [reference]). Statistically significant differences within each group over the years are denoted as: *p < .05, **p < .01, ***p < .001

Of the 74 programs that reported training extensions, the proportion requiring extensions declined steadily from 43.2% (32/74) in Year 1 to 33.8% (25/74) in Year 2 and 23.0% (17/74) in Year 3 (Fig. 4). This reduction was statistically significant (TM1; OR = 0.11, 95% CI: 0.03–0.44; *p* = .002). Among programs that required extensions, the number of affected trainees (TM2) varied, with a median of 1 trainee (IQR: 1–2.25) in Year 1 and 2 trainees (IQR: 1–2) in Years 2 and 3. Surgical programs were significantly less likely to report training extensions compared to hospital-based programs in both Year 2 and Year 3 (OR = 0.01, *p* = .027 and 0.029, respectively). No significant differences in TM1 were observed by income status or training type. Full subgroup results are provided in Supplementary Table 4.

Regarding platooning, 41.8% (59/141) of programs reported its use in Year 1 (TM3). While programs that implemented platooning had higher odds of reporting training extensions (OR = 4.00; 95% CI: 0.767–20.870), the association was not statistically significant, indicating no clear evidence of a relationship over time. Full detailed results for longitudinal trends and subgroup analyses are presented in Supplementary Tables 4a, 4b, 4c. 

## Discussion

This three-year longitudinal analysis highlights the ability of international residency and fellowship programs to endure the disruptions posed by the COVID-19 pandemic, with return to stable educational functioning without prolonged training extensions. Clinical experiences, which were most disrupted in Year 1, rebounded by Year 3. Non-elective educational program activities were preserved or rapidly adapted. Telemedicine use surged and its rapid integration reflected program flexibility in adopting new care modalities. Using a resilience engineering lens was helpful because it allowed these findings to be interpreted not simply as deficits or recovery, but as patterns of maintained function, adaptive substitution, and selective restoration over time. Within this framing, hybrid learning capacity refers to the ability of programs to preserve teaching through shifts in delivery modality, targeted remediation refers to focused educational responses where recovery remained incomplete, and digital equity refers to the infrastructural conditions that shape whether adaptive strategies can be implemented and sustained across settings. These patterns reflect principles of resilience engineering, which frame resilience as a system’s ability to adapt, evolve, and maintain function amid distress, rather than merely resisting or avoiding it.

Of note, all programs in this study had adopted CBME and successfully achieved accreditation from ACGME-I. The shared adoption of CBME provided a common framework with emphasis on flexibility, formative assessment, and tailored educational support - principles that may have contributed to institutional and programmatic resilience to continue meeting core competencies despite evolving clinical demands and shifting learning environments [6–8]. Similarly, a structured GME infrastructure with centralized oversight, coordinated support, and mechanisms to maintain supervision and teaching standards even under strain may have been relevant to the observed patterns [9]. Prior literature suggests that institutions with strong leadership and structured educational governance were more likely to sustain their investment in training and preserve core education functions, even amid resource constraints [10]. Our data are consistent with these dynamics at scale and over time, moving beyond early descriptive reports to show recovery patterns within a shared competency-based training framework.

Clinical training was predictably impacted, with ambulatory experiences showing the greatest reductions during the early phase of the pandemic. This likely resulted from the closure or repurposing of outpatient clinics, the rapid transition to telehealth, and public reluctance to seek non-urgent care [11–13]. Surgical specialties were particularly affected, with redeployments and suspension of elective procedures contributing to a significant reduction in operative volume [14], mirroring data from the United Kingdom [15]. However, our findings show that most surgical programs rebounded by Year 3 and were no more likely to report training extensions. This may be attributed to the timing of our study, which begins in 2021 after the initial phase of widespread cancellations, and therefore captures recovery-phase dynamics rather than peak disruption. It could also be explained by the rapid implementation of alternative strategies such as simulation-based learning and remote procedural training using virtual curricula [16, 17]. Importantly, urgent procedure exposure showed an incomplete return, indicating residual gaps that can guide targeted curricular supplementation rather than assuming full normalization.

The ability to maintain trainee progression despite fluctuating clinical structures underscores the flexibility embedded within competency-based program design and standards which encourage programs to rely on competency-based assessments rather than rigid time or volume thresholds when evaluating trainee readiness [18, 19]. Advancement decisions are based on achievement of milestones and entrustment decisions - a shift also seen in other regulatory bodies such as the American Board of Surgery, which accelerated the adoption of Entrustable Professional Activities (EPAs) to assess readiness for independent practice [20]. Our longitudinal observation that training extensions decreased steadily across years, representing one of the clearest indicators of recovery, supports the feasibility of progression decisions anchored in competence rather than time.

In contrast, clinical exposure in intensive care units (ICUs) and emergency departments (EDs) increased substantially in Year 1, as hospitals restructured to manage COVID-19 surges and address urgent staffing needs [21]. Structured educational requirements, including appropriate supervision and formal evaluations, may have helped ensure that these unplanned experiences retained high educational value [22] and may have enhanced resident competence in high-acuity care and interdisciplinary teamwork [23]. This capacity for adaptive reconfiguration and resource reprioritization is another hallmark of resilient training systems [24]. By Year 3, these surges largely receded toward baseline, aligning with a selective, rather than universal, reversion pattern.

The rapid uptake of telemedicine during the early phases of the pandemic was another area where program flexibility appeared important. Programs quickly adapted by training residents in remote communication, documentation, and virtual care delivery - skills that aligned with core competency domains, such as interpersonal communication and systems-based practice. Regulatory bodies provided temporary flexibility to facilitate this shift, enabling programs to integrate telehealth without delaying graduation [25]. Although the use of telemedicine declined as usual medical services resumed over time, approximately half of all programs continued to implement it for clinical care delivery by residents. Thus, telemedicine represents a durable, albeit attenuated, innovation rather than a transient stopgap, with implications for curricula, assessment, and infrastructure.

The adaptation to digital educational modalities was another pattern consistent with resilience. In-person didactics, were nearly universally canceled in the first year, prompting programs to adopt virtual platforms and hybrid instructional models, a format that became increasingly preferred by trainees [26]. Clear expectations for didactic activities, such as journal clubs, simulations, and multidisciplinary conferences allowed programs to replicate or adapt essential learning formats virtually, ensuring continued progress toward learning milestones [22]. Technology-enhanced learning methods have proven effective in building clinical skills, promoting self-directed learning, and enhancing communication and problem-solving abilities [27–30]. However, to sustain these gains, programs must invest in robust digital infrastructure, faculty development, and high-quality content [31]. This need is particularly relevant in lower-middle income countries, where virtual learning declined by Year 3, likely due to limited internet access, inadequate devices, and poor IT support, highlighting persistent concerns about digital inequity [32]. Our between-country analyses underscore these equity considerations and suggest that resilience is contingent on context and resources, not merely effort.

Another important dimension of resilience was the preservation of core educational relationships. Direct faculty supervision and advising remained stable throughout the three-year period, as did scholarly activity – an outcome relying on close resident-faculty interaction and mentoring. These relationships may have also offered vital support for psychological safety and learning continuity during uncertainty [33]. This continuity aligns with findings from Qatar, where improvements in the learning environment were noted in areas such as autonomy, social support, and teaching quality [34]. Similarly, in Singapore, the introduction of ACGME-I accreditation processes was associated with enhancements in educational infrastructure and the clinical learning environment [8]. Formal educational roles, including program directors and core faculty, likely promoted a shared sense of responsibility for resident development, helping sustain educator involvement even in the face of systemic disruption [34]. In combination, these features represent reproducible design elements (competency-based assessment, centralized oversight, faculty roles, and digital capacity) that other systems can adapt to bolster preparedness for future crises.

This study contributes to the literature by providing a longitudinal, multi-country analysis of GME program responses to the COVID-19 pandemic. While most prior studies offer cross-sectional or single-country perspectives, this research spans seven countries and encompasses a wide range of specialties and economic contexts. By capturing three consecutive years of data, the study reveals not only the immediate disruptions but also patterns of recovery and persistent gaps in clinical, educational, and telemedicine domains. It also identifies disparities in recovery trajectories by specialty and income setting, offering a more nuanced understanding of the resilience and adaptability of global GME systems. By applying a resilience lens longitudinally, the study extends earlier reports that primarily documented adaptation during the acute phase of COVID-19 and makes it possible to distinguish transient accommodations from more durable changes in training systems. This level of time-sensitive, comparative analysis represents a novel empirical contribution to the literature on medical education in times of crisis. Collectively, these findings advance the conversation from “what changed during COVID-19?” to “which changes endured, for whom, and under what conditions,” a shift that informs policy and accreditation decisions going forward.

A key interpretive insight is that the observed educational resilience appears linked to structured, well-supported training environments. Programs with strong institutional coordination, faculty development strategies, competency-based frameworks, and robust assessment systems were better able to absorb disruption while maintaining educational integrity. In resilience engineering terms, these programs demonstrated adaptive capacity - reconfiguring processes in real time and relying on established structures to guide decisions under pressure.

Yet these findings should be interpreted in light of certain limitations. First, the study only included ACGME-I accredited programs; locally accredited or unaccredited programs were excluded. Importantly, because all included programs operated within the same accreditation framework and no comparator group was available, causation cannot be determined. Second, data collection began in 2021, likely missing the earliest and most severe phases of disruption in 2020, particularly for procedure-based specialties. Accordingly, Year 1 (AY2021–22) should be interpreted as an already partially recovered baseline rather than the point of maximum disruption, and the magnitude of the initial pandemic-related effects is therefore likely underestimated. Third, reliance on self-reported, program-level data reduces the visibility of individual resident experiences and introduces potential for recall bias, social desirability bias, and a lack of detail regarding day-to-day educational realities. In addition, these program-level survey responses cannot confirm whether improvements in reported scores reflect true restoration of educational quality, as opposed to perceived recovery in structure, participation, or delivery. Fourth, the study does not include objective measurements of educational outcomes like skills acquisition, milestone progression, or board exam performance, making it difficult to assess the true educational efficacy of the adaptations described. Further, although the findings were stratified by specialty and income level, the study does not fully account for local contextual factors that influenced the severity of disruption, including public health policies, COVID-19 case burden, or sociopolitical instability. In addition, interactions between program characteristics (e.g., specialty and country income status) were not modeled. Exploratory analyses indicated that such models resulted in sparse data and unstable estimates, limiting interpretability. Lastly, because the study ends in 2024, it cannot evaluate the long-term sustainability of observed recovery trends or the enduring impact of the pandemic on medical education, such as burnout, faculty attrition, or ongoing curricular evolution. To fully understand the legacy of COVID-19 on global GME, future research should incorporate mixed methods, long-term longitudinal follow-up, as well as resident perspectives.

## Conclusion

This study provides a multi-year evaluation of international GME programs’ responses to the COVID-19 pandemic across diverse specialties, countries, and income settings under a common ACGME-I competency-based framework. Programs largely restored clinical and educational activities by Year 3 without widespread extensions, while maintaining supervision, advising, and scholarly engagement. The marked reduction in training extensions over time represents one of the clearest indicators of recovery across programs. Rather than a simple “back to normal,” our findings suggest a selective reversion with durable innovations, with hybrid didactics persisting, telemedicine attenuating but not disappearing, and urgent procedures showing an incomplete return. Recovery was heterogeneous by specialty and country income, underscoring that context and resources shaped trajectories.

What this adds beyond early pandemic reports is time-sequenced, multi-country evidence identifying which disruptions resolved, which practices persisted, and under what conditions, within a shared competency-based context. The findings are consistent with the hypothesis that elements such as competency-based assessment, centralized oversight, defined educator roles, and digital capacity, may enable resilience at scale, supporting progression decisions anchored in competence rather than time when clinical volumes fluctuate.

For policy and practice, the implication is to treat resilience as a designed property of training systems: protect core relationships (supervision/advising), preserve hybrid learning capacity, target remediation where residual gaps remain (e.g., urgent procedures), and invest in digital equity to avoid uneven recovery across settings. In our view, accreditation bodies and institutions could consider incorporating simple “resilience indicators” (e.g., continuity of direct observation, adaptability of didactics, telemedicine competency use-cases) into routine oversight to sustain gains beyond COVID-19 and prepare for future shocks. In sum, our longitudinal, multi-country analysis advances the conversation from “what changed during COVID-19?” to “which changes endured, for whom, and under what conditions,” providing actionable guidance to strengthen GME systems going forward.

## Supplementary Information

Supplementary File 1.

Supplementary File 2: Supplementary Table 1a.

Supplementary File 3: Supplementary Table 1b.

Supplementary File 4: Supplementary Table 1c.

Supplementary File 5: Supplementary Table 2a.

Supplementary File 6: Supplementary Table 2b.

Supplementary File 7: Supplementary Table 2c.

Supplementary File 8: Supplementary Table 3a.

Supplementary File 9: Supplementary Table 3b.

Supplementary File 10: Supplementary Table 3c.

Supplementary File 11: Supplementary Table 4a.

Supplementary File 12: Supplementary Table 4b.

Supplementary File 13: Supplementary Table 4c.

## Data Availability

The datasets used and/or analysed during the current study are available from the corresponding author on reasonable request.

## Abbreviations

ACGME-I: Accreditation Council for Graduate Medical Education International
AY: Academic year
CBME: competency-based medical education
CE: Clinical experiences
EDs: Emergency departments
EMM: Estimated marginal means
EP: Educational program activities
EPAs: Entrustable Professional Activities
GLMMs: Generalized linear mixed models
GME: Graduate medical educations
ICU: Intensive care units
IQR: Interquartile range
TM: Training modifications
TU: Telemedicine use
Y: Year
