# Supplementary material for: Pandemic to endemic: a longitudinal multinational study on COVID-19’s effects on graduate medical education
Source: BMC Med Educ. 2026 Jun 12;26:1335. doi: 10.1186/s12909-026-09657-y (PMC13487918; doi:10.1186/s12909-026-09657-y)
Supplement: Supplementary file 1 [file 12909_2026_9657_MOESM1_ESM.docx]

# **Supplementary file 1. Survey**

## **Domain 1. Program Characteristics**

1. Specialty:
   a. Medical
   b. Surgical
   c. Hospital
2. Training Type
   a. Residency
   b. Fellowship
3. Country
   a. Lebanon
   b. Oman
   c. Pakistan
   d. Qatar
   e. Saudi Arabia
   f. Singapore

*Note: In this study, the Country was recoded by Country Income Status, classified as lower-middle income or high income according to World Bank designations (2024).*

**The responses to the following domains should reflect what occurred in your program for this past academic year.**

**For each item, please indicate the overall effect of the pandemic over the past academic year, *compared to normal, pre-pandemic levels prior to March 2020*, on each of these activities for your *residents or fellows*:**

## **Domain 2. Clinical Experiences (CE)**

| **Activity** | Cancelled (0) | Significantly Decreased (1) | Decreased (2) | No Effect (3) | Increased (4) | Significantly Increased  (5) | Not Applicable |
| --- | --- | --- | --- | --- | --- | --- | --- |
| CE1. Ambulatory continuity clinic visits |  |  |  |  |  |  |  |
| CE2. Ambulatory-based clinical rotations/experiences |  |  |  |  |  |  |  |
| CE3. In-patient admissions |  |  |  |  |  |  |  |
| CE4. Urgent/emergent surgeries and/or procedures |  |  |  |  |  |  |  |
| CE5. Emergency department consultations |  |  |  |  |  |  |  |
| CE6. Intensive care unit coverage |  |  |  |  |  |  |  |

**Domain 3: Educational Program Activities (EP)**

| **Activity** | Cancelled (0) | Significantly Decreased (1) | Decreased (2) | No Effect (3) | Increased (4) | Significantly Increased  (5) | Not Applicable |
| --- | --- | --- | --- | --- | --- | --- | --- |
| EP1. In-person didactic conferences |  |  |  |  |  |  |  |
| EP2. Live virtual conferences |  |  |  |  |  |  |  |
| EP3. Elective rotations |  |  |  |  |  |  |  |
| EP4. Scholarly activity |  |  |  |  |  |  |  |
| EP5. Direct observation of residents/fellows by faculty |  |  |  |  |  |  |  |
| EP6. One-on-one advising sessions |  |  |  |  |  |  |  |

*Note: In this study, responses for Domains 2 and 3 (CE and EP) were recoded into a four-point ordinal scale to simplify analysis: Cancelled (0), Decreased (1) - combining Significantly and Moderately decreased, No effect (2), and Increased (3) - combining Moderately and Significantly increased. Not applicable (NA) responses were treated as missing.*

## **Domain 4: Telemedicine Use (TU)**

Regarding the use of telemedicine during the past academic year:

| **Activity** | None  (0) | 1-24% of patient  encounters  (1) | 25-49% of patient  encounters  (2) | 50-74% of patient  encounters  (3) | 75% or more of  patient encounters  (4) |
| --- | --- | --- | --- | --- | --- |
| TU1. What was the maximum use of telemedicine by your residents or fellows for providing clinical care? |  |  |  |  |  |
| TU2. How often was telemedicine used by faculty to supervise residents or fellows? |  |  |  |  |  |

## **Domain 5: Training Modifications (TM)**

**TM1. Were any residents or fellows required to extend their training for any reason related to the pandemic?**

| О Yes | О No |
| --- | --- |

**TM2. If yes, how many residents or fellows were required to extend their training for any reason related to the pandemic in [the previous academic year]?**

**TM3. During the past academic year, did you use a “platooning” system in your program due to the COVID-19 pandemic?**

*"Platooning" is a two or more team system that helps residents or fellows provide clinical care while ensuring the maintenance of a healthy workforce. Residents or fellows are divided into two groups: “active duty” and “working remotely.” Active duty residents or fellows participate in clinical care activities. Residents or fellows designated as working remotely participate in educational activities such as didactics, online modules, study, scholarly activities, and quality improvement projects. (choose one below).*

| О Yes | О No |
| --- | --- |
